# 5-Lypoxygenase Products Are Involved in Renal Tubulointerstitial Injury Induced by Albumin Overload in Proximal Tubules in Mice

**DOI:** 10.1371/journal.pone.0107549

**Published:** 2014-10-10

**Authors:** Sharon Schilling Landgraf, Leandro Souza Silva, Diogo Barros Peruchetti, Gabriela Modenesi Sirtoli, Felipe Moraes-Santos, Viviane Gomes Portella, João Luiz Silva-Filho, Carla Silva Pinheiro, Thiago Pereira Abreu, Christina Maeda Takiya, Claudia Farias Benjamin, Ana Acacia Sá Pinheiro, Claudio Canetti, Celso Caruso-Neves

**Affiliations:** 1 Instituto de Biofísica Carlos Chagas Filho, Universidade Federal do Rio de Janeiro, CCS, Rio de Janeiro, Rio de Janeiro, Brazil; 2 Instituto Federal de Educação, Ciência e Tecnologia, Rio de Janeiro, Rio de Janeiro, Brazil; 3 Instituto de Ciências Biomédicas, Universidade Federal do Rio de Janeiro, CCS, Rio de Janeiro, Rio de Janeiro, Brazil; 4 Departamento de Fisiologia e Biofísica, Instituto Nacional de Pesquisa Translacional em Saúde e Ambiente na Região Amazônica, CCS, Rio de Janeiro, Rio de Janeiro, Brazil; 5 Instituto Nacional de Ciência e Tecnologia em Biologia e Bioimagem, CCS, Rio de Janeiro, Rio de Janeiro, Brazil; Center for Molecular Biotechnology, Italy

## Abstract

The role of albumin overload in proximal tubules (PT) in the development of tubulointerstitial injury and, consequently, in the progression of renal disease has become more relevant in recent years. Despite the importance of leukotrienes (LTs) in renal disease, little is known about their role in tubulointerstitial injury. The aim of the present work was to investigate the possible role of LTs on tubulointerstitial injury induced by albumin overload. An animal model of tubulointerstitial injury challenged by bovine serum albumin was developed in SV129 mice (wild-type) and 5-lipoxygenase-deficient mice (5-LO^–/–^). The changes in glomerular morphology and nestin expression observed in wild-type mice subjected to kidney insult were also observed in 5-LO^–/–^ mice. The levels of urinary protein observed in the 5-LO^–/–^ mice subjected or not to kidney insult were lower than those observed in respective wild-type mice. Furthermore, the increase in lactate dehydrogenase activity, a marker of tubule damage, observed in wild-type mice subjected to kidney insult did not occur in 5-LO^–/–^ mice. LTB_4_ and LTD_4_, 5-LO products, decreased the uptake of albumin in LLC-PK1 cells, a well-characterized porcine PT cell line. This effect correlated with activation of protein kinase C and inhibition of protein kinase B. The level of proinflammatory cytokines, tumor necrosis factor-α and interleukin (IL)-6, increased in mice subjected to kidney insult but this effect was not modified in 5-LO^–/–^ mice. However, 5-LO^–/–^ mice subjected to kidney insult presented lower macrophage infiltration and higher levels of IL-10 than wild-type mice. Our results reveal that LTs have an important role in tubulointerstitial disease induced by albumin overload.

## Introduction

Leukotrienes (LTs) are lipid mediators derived from the metabolism of arachidonic acid by the action of 5-lipoxygenase (5-LO) in the presence of 5-LO activating protein (FLAP) [1]. The major biologically active products in this pathway are LTB_4_ and the cysteinyl-LTs (LTC_4_, LTD_4_, and LTE_4_) [1]. The role of LTs in renal physiology and pathophysiology has been shown in different animal models [2]. 5-LO mRNA has been found in leukocytes [3], dendritic cells [4], smooth muscle cells [5], and areas of the kidney, including the cortex, outer medulla, and inner medulla [6]. In addition, LTs are powerful chemotactic molecules, increasing leukocyte migration and activation [7]. In addition, Moore et al. [8] showed that kidney tissue can produce LTs independent of the circulating cells.

Several authors have pointed out the role of LTs in glomerular injury in different renal diseases [9]. However, little is known about the role of 5-LO products in tubulointerstitial injury, and consequently, in the progression of renal disease. Clinical investigations have demonstrated that 5-LO activity and expression are increased in peripheral blood mononuclear cells in patients with renal disease [10]–[12]. The inhibition of leukotriene biosynthesis improves renal function in different experimental models such as glomerulonephritis, renal ischemia–reperfusion and cyclosporine-induced nephrotoxicity [13]–[19]. Hagar and Tawab [16], studying a rat model of renal ischemia–reperfusion, showed that the cysteinyl leukotriene receptor blocker, zafirlukast, reduced the severity of ischemic acute renal failure via an anti-inflammatory action.

There is a strict correlation between proteinuria and a high risk of progression of renal disease [20], [21]. In part, this correlation is due to the fact that the overload of albumin in proximal tubule (PT) cells induces proinflammatory and profibrotic effects and contributes directly to the development of tubulointerstitial injury, and, consequently to progression of renal disease [20]. Albumin overload in PT cells has been show to induce the secretion of RANTES (regulated on activation, normal T cell expressed and secreted), monocyte chemotactic protein-1 (MCP-1), interleukin (IL)-6, IL-8 and transforming growth factor (TGF)-β [20], [22]. The molecular mechanisms underlying this process involve inhibition of the phosphoinositide-3-kinase (PI-3K)/protein kinase B (PKB) pathway and activation of NF-κB (nuclear factor kappa-light chain enhancer of activated B cells) [23]. Thomas et al. [24] showed that during tubulointerstitial injury, an increase in apoptosis of PT cells occurs. In this context, our group has shown that albumin overload in PT cells leads to inhibition of the PI-3K/PKB pathway, which is correlated to induction of apoptosis in LLC-PK1 cells with megalin as the sensor in this process.

In the present work, we studied the role of LTs in the development of tubulointerstitial injury induced by albumin overload. An animal model of tubulointerstitial injury induced by bovine serum albumin (BSA), a well-known *in vivo* model used to induce tubulointerstitial disease, was developed in SV129 (wild-type) and 5-LO^–/–^ mice [25], [26]. Our data revealed that 5-LO products are important mediators of tubulointerstitial injury induced by albumin overload. This effect seems to suppress the secretion of the anti-inflammatory cytokine IL-10 and inhibit albumin uptake mediated by inhibition of the PI-3K/PKB pathway and activation of protein kinase C (PKC).

## Materials and Methods

### Animals

We used a male 129-Alox5tm1Fun/J (5-LO-deficient; 5-LO^−/−^) mouse and its SV129 littermates as controls (wild-type; WT), which according to the new nomenclature that has been approved by the Committee on Standardized Genetic Nomenclature for Mice, is now called 129S1/SvImJ. These mice are complete knockout. We have previously used these animals in several studies, using different read outs, and we test them (twice a year) for 5-LO protein expression (by Western blot) and LT production (using EIA kits, Cayman, Ann Arbor, MI) in order to confirm the knockout status. Animal procedures were conducted in accordance with the National Institutes of Health (NIH) Guide for the Care and Use of Laboratory Animals and were approved by the Institutional Ethics Committee of Federal University of Rio de Janeiro (number IBCCF004).

### Experimental Protocol

An animal model of tubulointerstitial injury induced by BSA was developed as described previously [25], [26]. Briefly, animals weighing 20–22 g were randomly divided into 4 groups, subjected to intraperitoneal (i.p.) injections of saline (0.9%) or 10 g/kg BSA for 7 consecutive days: (1) SV129 mice treated with saline (WT+SAL); (2) SV129 mice treated with 10 g/kg BSA (WT+BSA); (3) 5-LO^−/−^ mice treated with saline (5-LO^−/−^+SAL); and (4) 5-LO^−/−^ mice treated with 10 g/kg BSA (5-LO^−/−^+BSA). At least 6 animals were used in each group. Treatment for 7 days was chosen because all the injuries observed in this animal model have developed by this time [27]. During treatment, the animals were housed in metabolic cages as described below. At the end of the treatment (day 7), the mice were anesthetized with ketamine (80 mg/kg body weight) and xylazine (5 mg/kg body weight); blood samples were collected by cardiac puncture and the kidneys were removed. One kidney was used for immunohistologic studies and the other kidney was used to prepare the homogenate fraction of the kidney cortex, as described below.

### Basic features of the BSA-induced tubulointerstitial injury model

**Figure 1:**
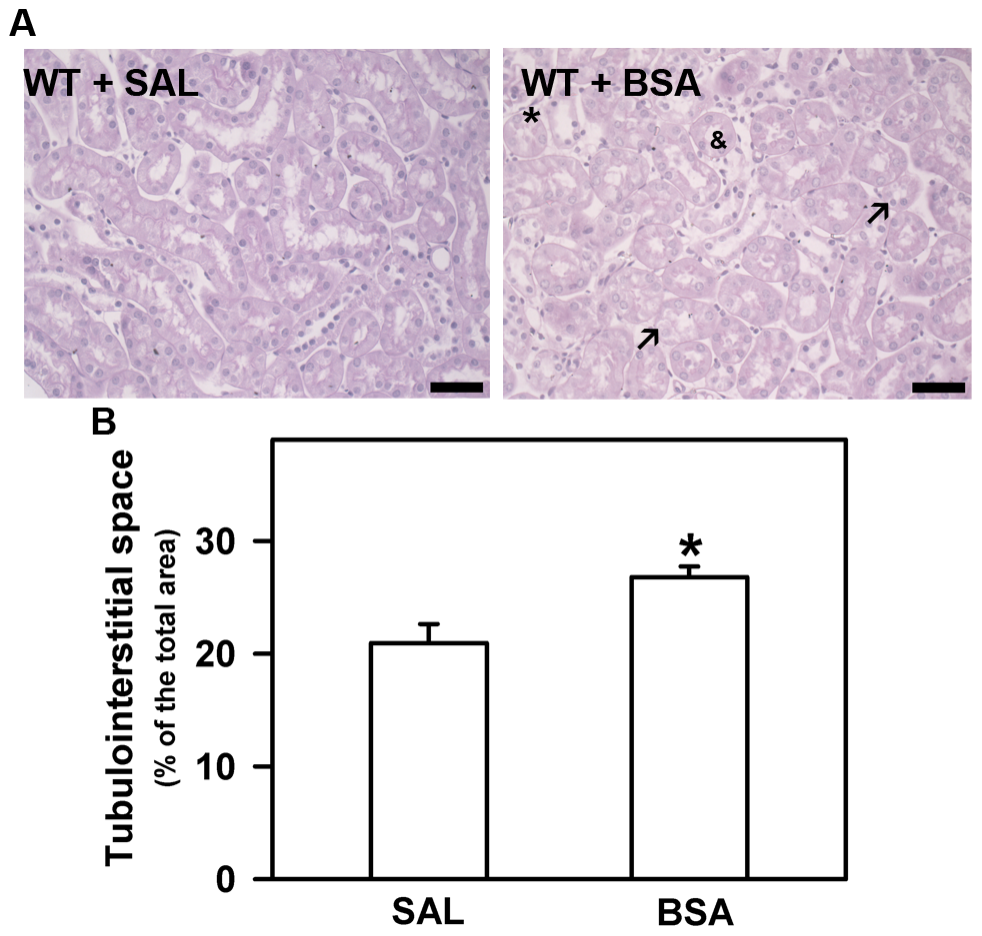
Basic features of BSA-induced tubulointerstitial injury model. (A) Representative Periodic-acid Schiff staining in renal cortex of WT+SAL and WT+BSA groups. Arrows, indicates epithelial cell vacuolization; *, indicates tubular shedding; &, indicates disorganization of brush-border. (B) Quantification of tubulointerstitial space area. Results were expressed as means ± SE. Statistically significant in relation to *WT + SAL (*p*<0.05).

**Figure 2:**
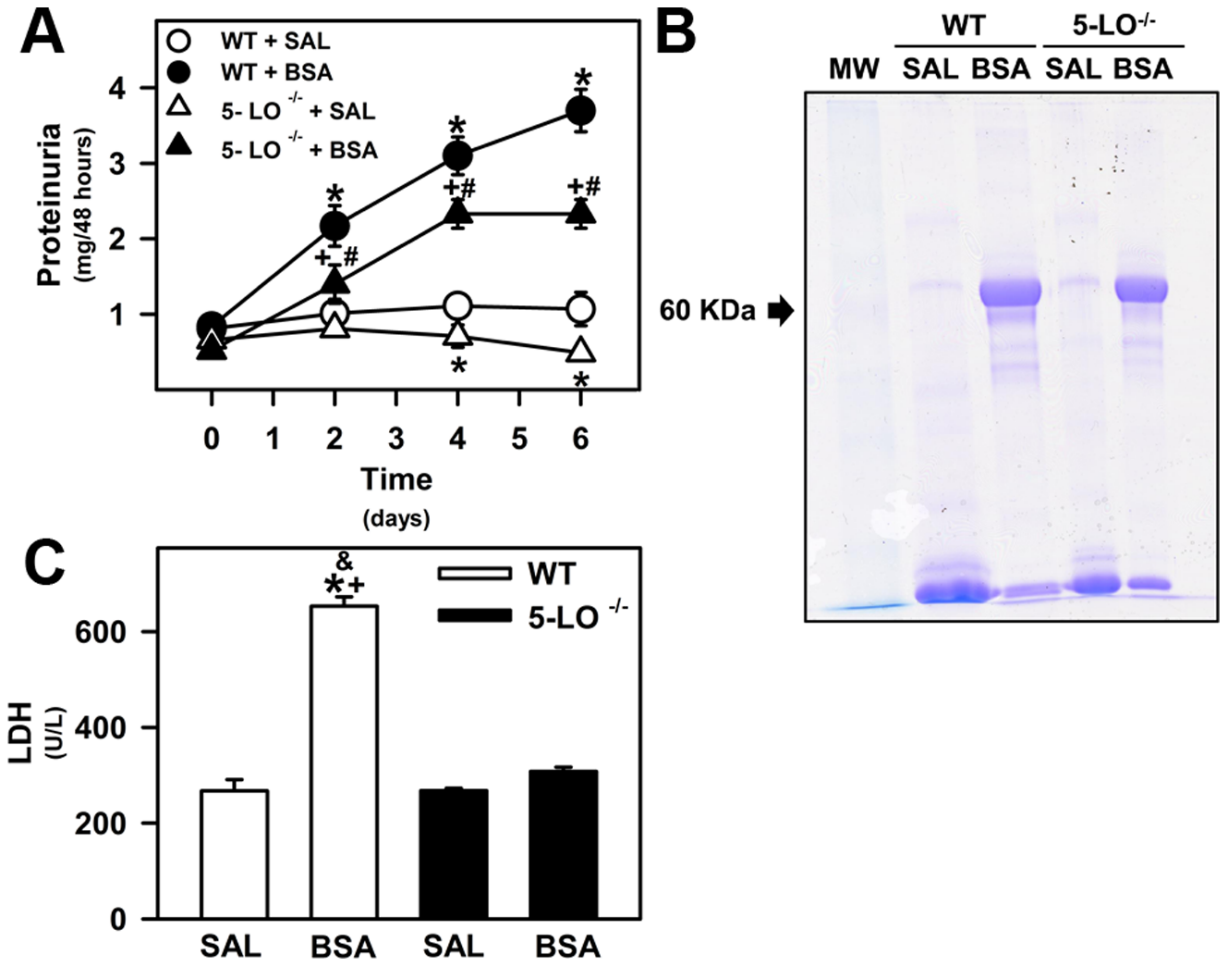
Proteinuria and urinary tubular enzymes are attenuated in 5-LO-deficient mice subjected to kidney injury. The animals were given i.p. injections of saline (SAL; 0.9%) or 10 g/kg BSA for 7 days as described in the Materials and Methods section. (A) The proteinuria was measured on days 0, 2, 4, and 6. (B) Urine samples were resolved on SDS-PAGE gels and protein analysis was based on the intensity of Coomassie Blue staining. (C) LDH was measured in urine samples as an index of cell damage. WT+SAL (*n* = 8), WT+BSA (*n* = 8), 5-LO^–/–^+SAL (*n* = 8), 5-LO^–/–^+BSA (*n* = 8). The results are expressed as means ± SE. Statistically significant in relation to *WT+SAL (*p*<0.05), #WT+BSA (*p*<0.05), ^+^5-LO^–/–^+SAL (*p*<0.05), and ^&^5-LO^–/–^+BSA (*p*<0.05).

This model was developed previously using C57BL/6 or SV129 mice strains [28]. An increase in the glomerular permeability to protein, albumin overload in PT and proteinuria were observed. In addition, this scenario was associated with tubulointerstitial macrophage infiltration in the renal cortex. In a previous study using BALB/c mice, we found shedding, vacuolization, disorganization of the brush border in cortical epithelial cells, and an increase in the area of tubulointerstitial space associated with increase in urinary LDH activity [26], [29]. In order to highlight these pathologic features of the BSA-induced tubulointerstitial injury model, morphologic changes in the cortex were assessed. Tubular injury in the WT+BSA group was characterized by disorganization of the apical border, shedding of epithelial cell membrane, vacuolar degeneration, increased area of tubulointerstitial space by examination of 15 randomly selected fields from each animal at ×40 magnification (high quality images 2048×1536 pixels), as previously described (Figure 1). In addition, urinary LDH activity and proteinuria were also evaluated (Figure 2).

### Renal Function Analysis

The animals were housed individually in metabolic cages before treatment (day 0); renal function was analyzed on days 2, 4, 6, and 7. The cages were maintained in a temperature-controlled room regulated on a 12-h light/dark cycle, and the animals had free access to standard chow and tap water. Urine samples were collected for 48 h and used to determine urinary volume, urinary protein, lactate dehydrogenase (LDH), and creatinine concentration. Before urinary analysis, the samples were centrifuged at 3000×*g* for 5 min and the supernatant was separated and stored at –20°C until the assays. The levels of protein were determined by the Pyrogallol Red method (Gold Analisa kit no. 498M, Belo Horizonte, MG, Brazil) and creatinine was measured using the alkaline picrate method (Gold Analisa kit no. 335). A kit for LDH (Gold Analisa kit no. 457) was used for quantitative determination of the enzyme activity. Blood samples were centrifuged at 2000×*g* for 10 min to obtain plasma to measure the creatinine concentration.

### Preparation of the Homogenate Fraction

The homogenate fraction of the renal cortex was obtained as described previously [30]. Briefly, the kidney were removed and homogenized in a cold solution containing 250 mmol/L sucrose, 10 mmol/L HEPES–Tris (pH 7.6), 2 mmol/L EDTA, and 1 mmol/L phenylmethylsulfonyl fluoride. The homogenate was centrifuged at 7000×*g* at 4°C for 10 min and the final supernatant was stored at −80°C. Protein concentrations were determined by the Folin phenol method [31] using BSA as standard.

### Cell Culture and Biochemical Assay

LLC-PK1 cells, a well-characterized porcine PT cell line, were obtained from American Type Culture Collection (Rockville, MD). The cells were maintained in low-glucose Dulbecco's modified Eagle's medium with 10% fetal bovine serum/1% penicillin/streptomycin (37°C and 5% CO_2_) [23]. The cells were used after confluence was reached, typically 3 days after seeding. When indicated, the cells were preincubated overnight with different LTs (10^−7^ M LTB_4_ or 10^−7^ M LTD_4_) in serum-depleted medium. Albumin uptake and protein kinase activities were then measured as described [32], [33].

### Albumin Uptake

The LLC-PK1 cells were incubated with Ringer solution containing 15 µg/mL fluorescein isothiocyanate (FITC)-BSA at 37°C for 15 min (for endocytosis). Unbound FITC-albumin was removed by rinsing 8 times with ice-cold Ringer solution. Cells were lysed using detergent (0.1% Triton X-100 in 4-morpholinopropanesulfonic acid (MOP) solution) and the cell-associated fluorescence was measured using a microplate spectrofluorometer (SpectraMax M2, Molecular Devices, Sunnyvale, CA).

### Immunoblotting

Phosphorylated and total PKB were immunodetected in the homogenate fraction of renal cortex with specific primary antibodies (1∶1000, Cell Signaling Technology, Danvers, MA, catalog nos. 9271 and 9272, respectively). Proteins were resolved on sodium dodecyl sulfate (SDS)-polyacrylamide gel electrophoresis (PAGE) gels and transferred to polyvinylidene difluoride (PVDF; Amersham Biosciences, Piscataway, NJ) according to the manufacturer's instructions. After antibody labeling, detection was performed using ECL Plus (Amersham Biosciences, Piscataway, NJ).

### Measurement of Protein Kinase Activity

The activity of PKC and protein kinase A (PKA) was measured by incorporating ^32^Pi from [γ^32^-P]ATP (7 mCi/mmol) into histone sensitive to 10^−8^ mol/L calphostin C (Calbiochem, Billerica, MA; catalog no. 208725) and 10^−8^ M PKA inhibitor peptide (PKAi; Sigma-Aldrich, St Louis, MO; catalog no. B1427), respectively [30], [34]. The composition of the reaction medium was 4 mM MgCl_2_, 20 mM HEPES–Tris (pH 7.0), 1.5 mg/mL histone, and 0.7 mg/mL protein. The reaction was stopped with 30% trichloroacetic acid (TCA) and the sample was immediately placed on ice. An aliquot (0.1 mL) was filtered through a Millipore filter (0.45 mm; Millipore, Billerica, MA; catalog no. HAWP29325) and washed with ice-cold 20% TCA solution and 0.1 M phosphate buffer (pH 7.0). The radioactivity was quantified by liquid scintillation counting (Packard Tri-Carb 2100 TR). The specific PKC and PKA activities were calculated from the difference between the activity in the absence and in the presence of 10^−8^ M calphostin C and 10^−8^ M PKAi, respectively. Phorbol myristate acetate (PMA) was used as PKC activator and cyclic adenosine monophosphate (cAMP) was used as PKA activator.

### Histological and Immunohistochemical Analysis

Kidneys were fixed in a 4% buffered formalin solution and embedded in paraffin. Histologic sections (3-µm thick) of kidney were obtained and stained with periodic acid-Schiff reagent (Sigma-Aldrich, St Louis, MA). Total collagen staining in renal cortical slices was assessed using Picrosirius Red. The glomerular tuft and mesangial surface of subcapsular and corticomedullary glomeruli were measured and determined using Image-Pro Plus analysis software. Fifteen glomeruli per animal were captured at 400× magnification (high-quality images 2048×1536 pixels) and then analyzed. Renal macrophages were characterized by reactivity for the F4/80 antibody (1∶50; AbD Serotec, Raleigh, NC; catalog no. MCA497), cortical total TGF-β was determined by reactivity for the pan-TGF- β antibody (1∶50, R&D Systems, Minneapolis, MN), followed by the Histofine SimpleStain Mouse secondary antibody (Nichirei Biosciences, Tokyo, Japan; catalog no. 414311F) and revealed using the chromogen diaminobenzidine (liquid DAB, DAKO, Carpinteria, CA; catalog no. K3468). The expression of glomerular nestin was also determined by immunohistochemical staining using an anti-nestin primary antibody (Chemicon, Temecula, CA). The cortical and medullary macrophage content and the nestin density were assessed using image analysis software (Image-Pro Plus). Fifteen microscopic fields of interstitial areas and glomeruli per animal were randomly captured to obtain the surface density of F4/80 reactivity, and nestin, respectively. All histologic analyses were conducted in blind fashion.

### Measurement of Renal Cytokines

The concentration of tumor necrosis factor (TNF)-α, IL-6, and IL-10 in the kidneys was evaluated by ELISA (BD Biosciences), according to the manufacturer's instructions. The results are expressed as pg/mL of protein.

### Statistical Analysis

The results are expressed as means ± standard error (SE). Differences between the control and experimental groups were analyzed by one-way analysis of variance followed by the Newman–Keuls test for multiple comparisons.

## Results

### Deficiency of 5-LO Attenuates Proteinuria in an Animal Model of Tubulointerstitial Injury Induced by BSA

To evaluate the role of 5-LO products during tubulointerstitial injury, WT and 5-LO^–/–^ mice were treated with BSA (10 g/kg per day) or saline for 7 days (WT+BSA, 5-LO^–/–^+BSA, WT+SAL, and 5-LO^–/–^+SAL, respectively), as described in the Materials and Methods section. Body weight, 48-h water intake, 48-h urine flow, plasma/urinary creatinine, and creatinine clearance (CCr) were measured at baseline (day 0) and at the end of treatment (day 7), as shown in Table 1. At day 0, these parameters were not different between WT and 5-LO^–/–^ mice. At day 7, the 48-h urine flow was higher in all BSA-treated groups (WT+BSA, 5-LO^–/–^+BSA) than in the saline groups (WT+SAL, 5-LO^–/–^+SAL). There was no difference in urine flow between the WT and 5-LO^–/–^ mice. However, BSA treatment had no major effect on the other parameters analyzed (body weight, water intake, plasma/urinary creatinine, and CCr) in both WT and 5-LO^–/–^ mice (Table 1).

**Table 1:** Renal function parameters.

	WT+SAL		WT+BSA		5-LO^–/–^+SAL		5-LO^–/–^+BSA	
	Day 0	Day 7	Day 0	Day 7	Day 0	Day 7	Day 0	Day 7
Body weight (g)	21.7±1.40	20.4±0.89	21.6±0.52	21.6±0.50	21.4±0.63	20.9±0.71	21.7±0.52	21.7±0.52
48-h water intake (mL)	7.08±1.19	6.20±0.98	6.94±0.36	6.30±0.36	7.75±0.74	6.90±0.79	7.81±0.61	7.10±1.08
48-h urinary flow (µL/min)	0.41±0.04	0.49±0.07	0.45±0.06	0.83±0.09*	0.48±0.05	0.54±0.05	0.54±0.10	0.84±0.08^+^
Plasma creatinine (mg/dL)	0.25±0.02	0.24±0.04	0.25±0.03	0.26±0.03	0.24±0.07	0.26±0.06	0.29±0.04	0.28±0.05
Urinary creatinine (mg/dL)	58.4±8.31	45.1±3.90	57.0±6.80	35.5±2.61	60.6±7.45	48.2±4.93	61.6±14.8	39.4±2.82
Creatinine clearance (µL/min)	103.3±23.7	118.5±12.1	125.7±15.5	119.4±17.6	117.0±10.8	116.6±23.3	113.5±19.8	119.2±16.2

Statistically significant in relation to *WT+SAL (day 7; *p*<0.05), ^+^5-LO^–/–^+SAL (day 7; *p*<0.05).

We also measured the amount of protein excreted in 48 h, a marker of renal injury, in the urine of WT and 5-LO^–/–^ mice at day 0 and after initiation of treatment (days 2, 4, and 6). At day 2, all BSA-challenged groups (WT and 5-LO^–/–^) presented higher urinary protein excretion than their respective controls and this profile was sustained until the end of the study (Figure 2A). The magnitude of the proteinuria response to kidney insult in 5-LO^–/–^ mice was lower than that observed in WT mice (Figure 2A, B). 5-LO^–/–^+SAL mice had lower levels of proteinuria (0.58±0.07 mg/48 h) compared with WT+SAL mice (1.15±0.15 mg/48 h) (Figure 2A).

LDH activity was also measured in the urine of WT and 5-LO^–/–^ mice treated with saline or BSA, as an index of cell damage. LDH activity was increased in the WT+BSA group, but it was not changed in the 5-LO^–/–^+BSA group (Figure 2C). These results show that 5-LO products contribute to the hyperproteinuric state and renal tissue damage profile observed in mice subjected to kidney insult.

**Figure 3:**
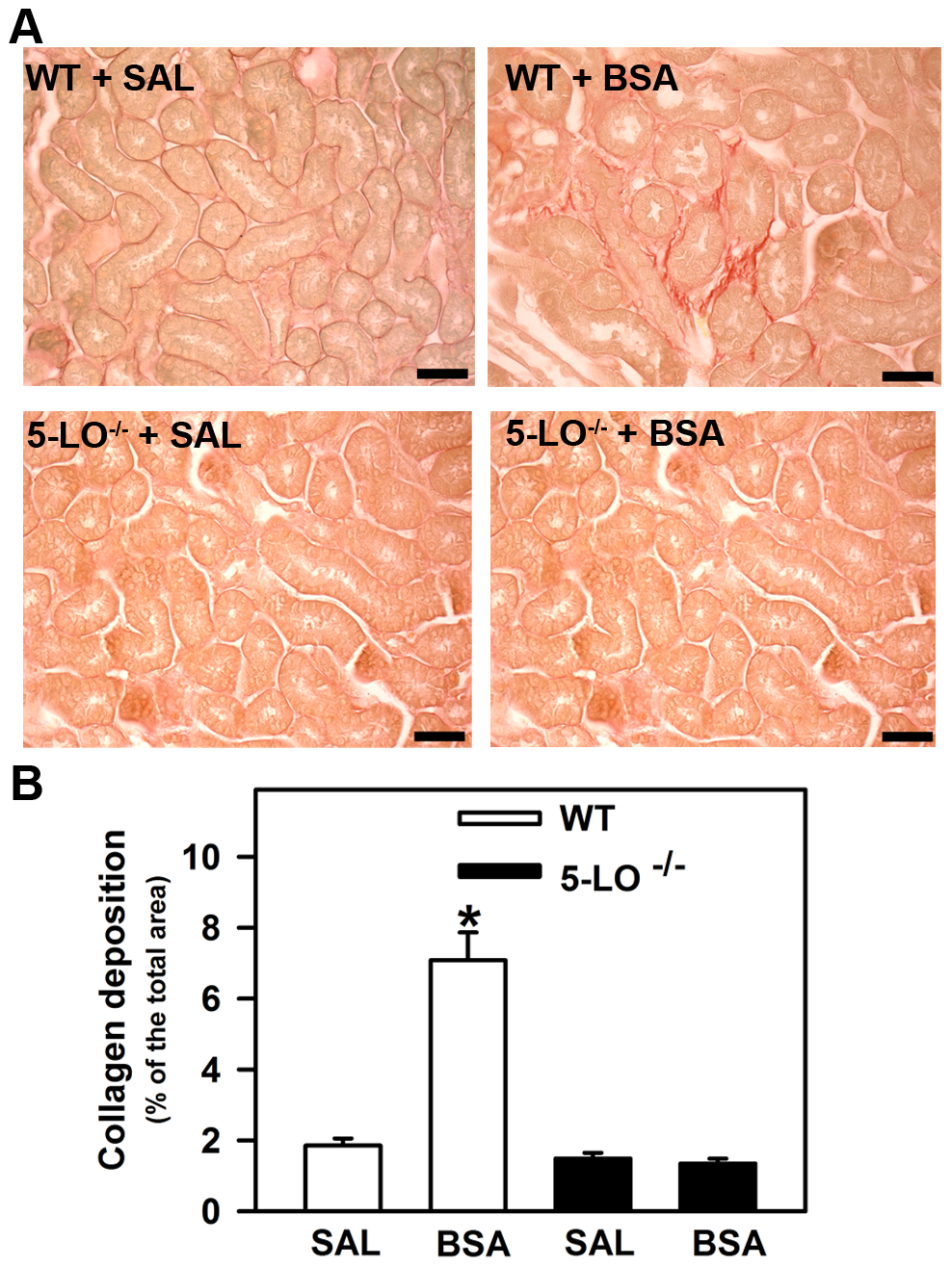
The effect of 5-LO products on total cortical collagen deposition induced by kidney injury. Mice were treated as described in Materials and Methods section (*n* = 6 per group). (A) Representative PicroSirius staining in cortical area of WT mice and 5-LO-deficient mice treated with saline or BSA (bars  = 40 µm). (B) Quantitative analyses were expressed as means ± SE. Statistically significant in relation to *WT + SAL (*p*<0.05).

Usually, renal diseases that are followed by loss of protein in the urine lead to the presence of kidney fibrosis [22], [28], [29]. Thus, we verified collagen deposition in mice treated with BSA and whether there was any correlation with 5-LO expression. An increase in renal cortex collagen deposition was observed in the WT+BSA group (Figure 3). This increase in collagen deposition was not observed in 5-LO^–/–^ animals subjected to tubulointerstitial injury (5-LO^–/–^+BSA) (Figure 3).

### 5-LO-Derived Products Are Not Involved in Glomerular Injury

**Figure 4:**
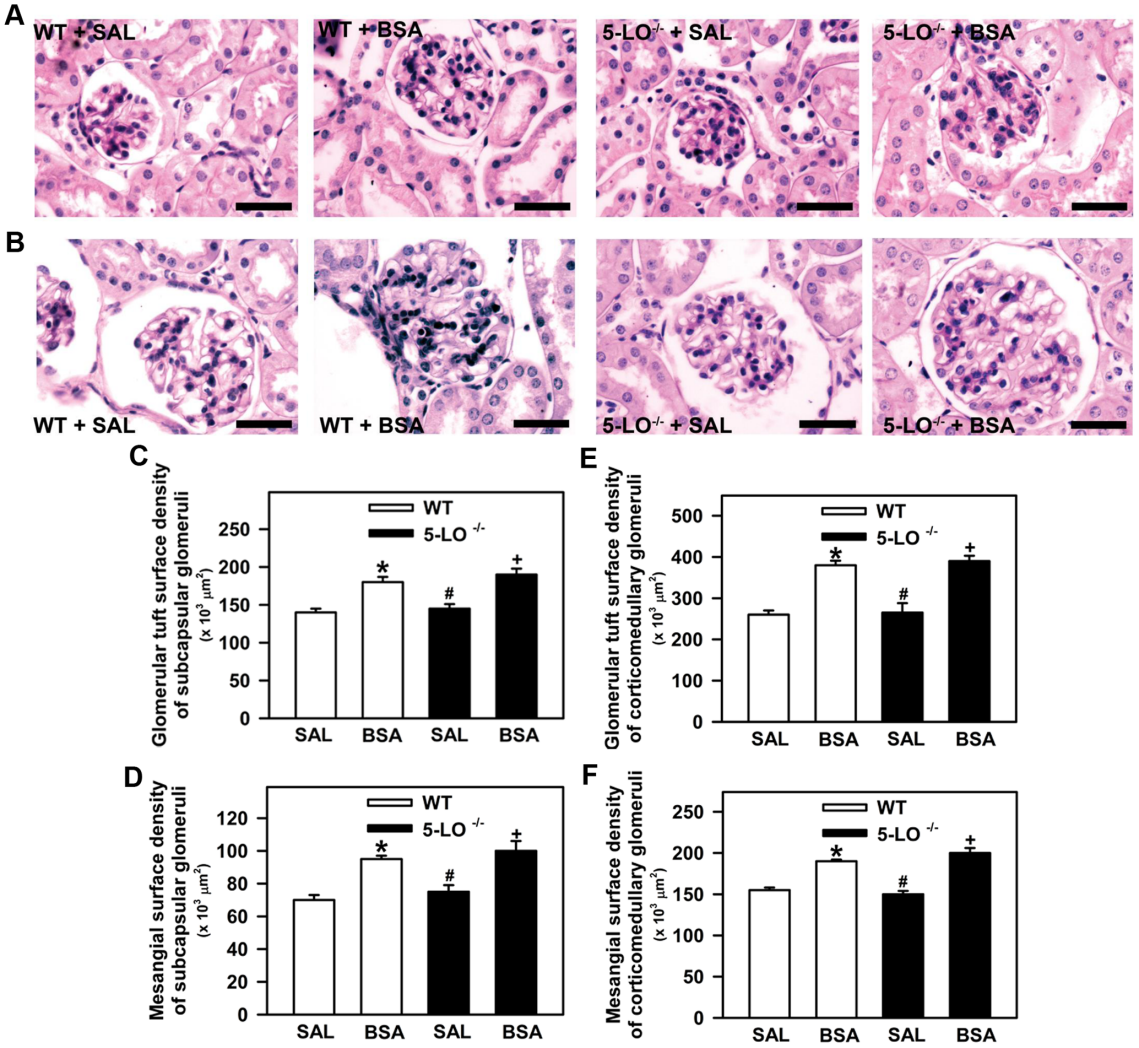
Glomerular morphology in WT and in 5-LO-deficient mice after 7 days of treatment with saline or BSA. Mice were treated as described in Fig. 1 (*n* = 6 per group). Kidney sections were stained with periodic acid–Schiff. (A) Representative photomicrographs of the subcapsular glomeruli (bar  = 40 µm) and (B) corticomedullary glomeruli (bar  = 40 µm). Quantitative analysis of (C) the glomerular tuft surface of the subcapsular glomerulus, (D) the glomerular tuft surface of the corticomedullary glomerulus, (E) the mesangial surface of the subcapsular glomerulus, and (F) the mesangial surface of the corticomedullary glomerulus. The results are expressed as means ± SE. Statistically significant in relation to *WT+SAL (*p*<0.05), #WT+BSA (*p*<0.05), ^+^5-LO^–/–^+SAL (*p*<0.05).

**Figure 5:**
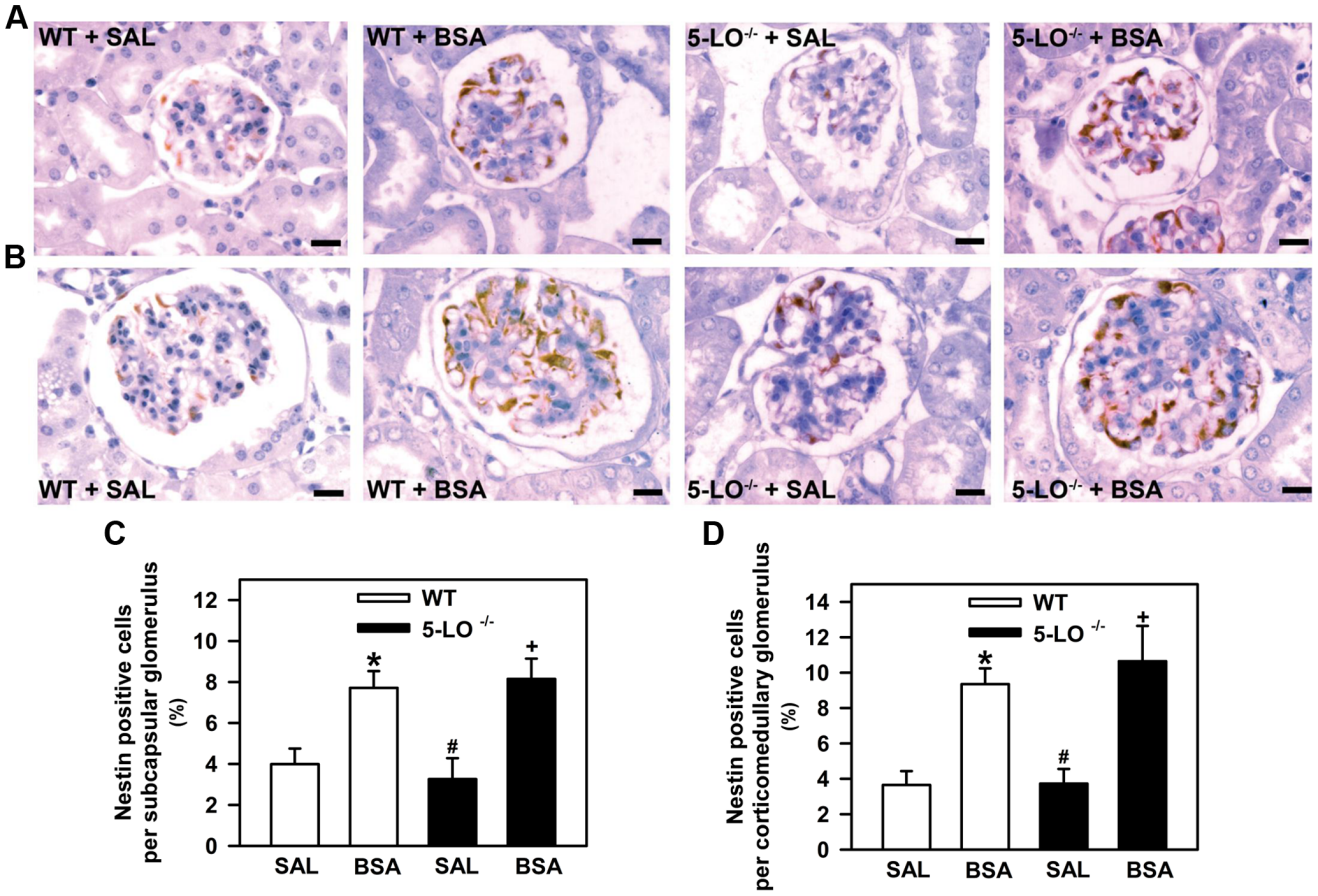
Nestin expression is increased in subcapsular and corticomedullary glomeruli of mice subjected to kidney injury. Mice were treated as described in Fig. 1 (*n* = 6 per group). (A) Representative immunohistochemical staining for nestin in the subcapsular glomerulus and (B) corticomedullary glomerulus of WT and 5-LO-deficient mice treated with saline or BSA (bars  = 20 µm). Quantitative analyses (C,D) were expressed as means ± SE. Statistically significant in relation to *WT+SAL (*p*<0.05), #WT+BSA (*p*<0.05), ^+^5-LO^–/–^+SAL (*p*<0.05).

In this experimental setup, we investigated whether the hyperproteinuric profile observed in mice treated with BSA was associated with glomerular injury. Glomerular tuft, mesangial surface, and nestin expression were determined. It is well known that nestin, an intermediate filament protein, plays an important role in the maintenance of normal podocyte function [35]. Therefore, nestin expression was used as an index of podocyte injury [35]. These histologic parameters were assessed at the end of the study by histomorphometric analysis and immunohistochemistry, as described in the Materials and Methods section. The glomerular tuft surface of subcapsular (Figure 4A, C) and corticomedullary glomeruli (Figure 4B, D) was higher in the BSA groups than in the SAL groups. Similar to the glomerular tuft, the mesangial surface of subcapsular and corticomedullary glomeruli was increased in mice subjected to BSA treatment compared with the saline groups (Figure 4E, F). Both glomerular parameters measured were not different between the WT+BSA and 5-LO^–/–^+BSA groups. The expression of nestin in subcapsular (Figure 5A, C) or corticomedullary glomeruli (Figure 5B, D) was the same for 5-LO^–/–^+SAL and WT+SAL mice. On the other hand, glomerular nestin expression in subcapsular or corticomedullary glomeruli was increased in all BSA-challenged groups (WT+BSA or 5-LO^–/–^+BSA) compared with their respective saline groups (WT+SAL or 5-LO^–/–^+SAL). However, there were no differences between the BSA-challenged groups in WT and 5-LO^–/–^ mice. These results indicate that changes in glomerular permselectivity could explain the hyperproteinuric profile observed in BSA-challenged groups. However, the lower levels of proteinuria observed in 5-LO^–/–^ mice are not due to differences in glomerular permselectivity.

Looking for the mechanism involved in the decrease in proteinuria observed in 5-LO^–/–^ mice, we decided to investigate the modulation of albumin reabsorption in PT cells.

### LTs Modulate Albumin Uptake in PT Cells

**Figure 6:**
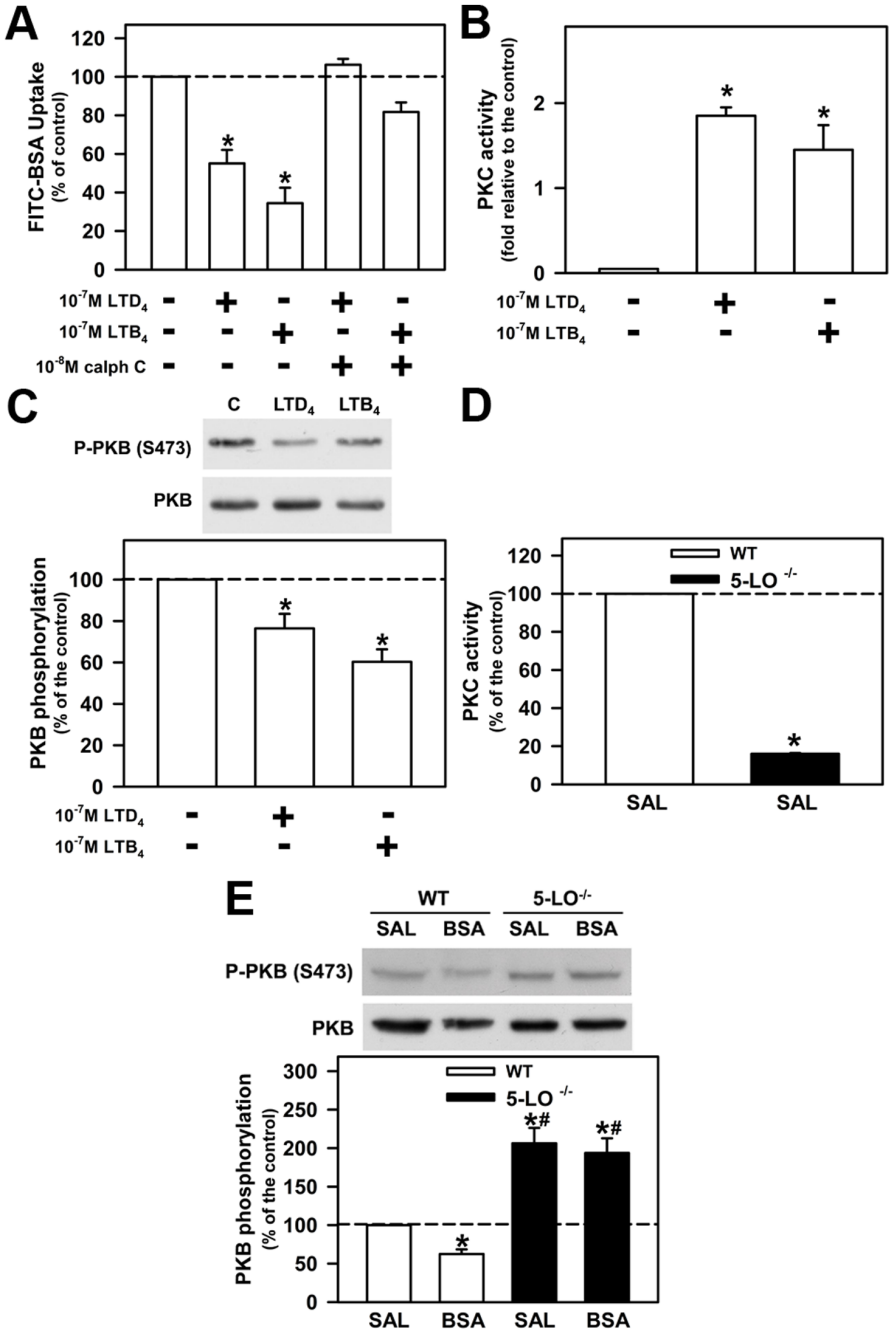
LTs modulate albumin uptake and kinase activity. LLC-PK1 cells were grown on 6-well plates, kept overnight in medium depleted of serum in the presence of 10^−7^ M LTD_4_ or 10^−7^ M LTB_4_. After treatment, both (A) albumin uptake and (B) PKC activity were measured as described in the Materials and Methods section. The cells were preincubated with 10^−8^ M calphostin C (Calph C) when indicated. (C) The effect of LTs on PKB activity measured by Ser473 phosphorylation. PKC (D) and PKB (E) activities were measured in WT and 5-LO-deficient mice treated with saline or BSA. The results are expressed as means ± SE. Statistically significant in relation to *control or WT+SAL (*p*<0.05), #WT+BSA (*p*<0.05).

LT receptors are linked to trimeric G protein and, consequently, they could change different serine/threonine kinase activities [7]. Furthermore, it is well known that albumin endocytosis is modulated by serine/threonine kinases such as PKB and PKC [33], [36]. Therefore, we decided to investigate if 5-LO products, LTB_4_ and LTD_4_, modulate albumin endocytosis in PT cells and the possible involvement of PKB and PKC. To address this question, LLC-PK1 cells, a well-known PT cell model, were incubated overnight with 10^−7^ M LTB_4_ or LTD_4_, in the presence or in the absence of PKC inhibitor, 10^−8^ M calphostin C. Albumin uptake was decreased by 10^−7^ M LTB_4_ and 10^−7^ M LTD_4_ (Figure 6A). This effect was completely abolished by PKC inhibitor at 10^−8^ M. Both LTs were found to stimulate PKC activity (Figure 6B), whereas the activity of PKB, measured by phosphorylation of serine residue 473 (Ser473), decreased (Figure 6C). Furthermore, the activity of PKB and PKC was measured in a cortical preparation of WT and 5-LO^–/–^+SAL mice. The PKC activity was decreased in 5-LO^–/–^+SAL mice compared with WT mice (Figure 6D), whereas the activity of PKB increased (Figure 6E). At this point, it is relevant to point out that the inhibitory effect of PKB activity observed in the WT+BSA group was not observed in the 5-LO^–/–^+SAL group. These data indicate that 5-LO products could be mediating the inhibitory effect of PKB activity by protein overload in PTs.

### 5-LO^–/–^ Mice Show a Marked Reduction in Interstitial Macrophage Infiltration

**Figure 7:**
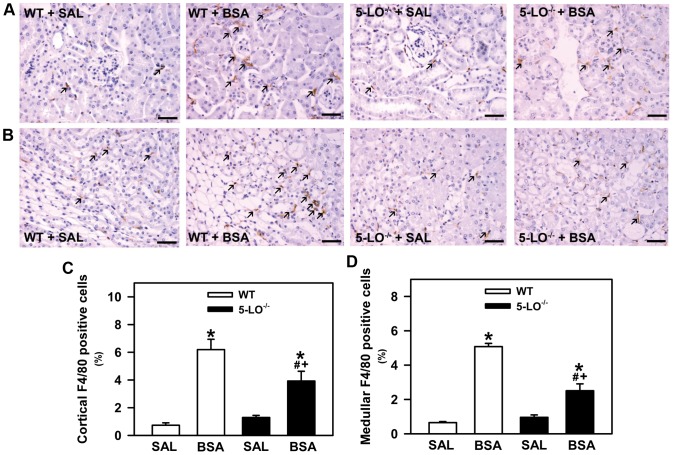
Macrophage infiltration is attenuated in 5-LO-deficient mice subjected to kidney injury. Mice were treated as described in Fig. 1 (*n* = 6 per group). Representative immunohistochemical staining for F4/80 in (A) cortical and (B) medullary areas of WT mice and 5-LO-deficient mice treated with saline or BSA (bars  = 40 µm). Quantitative analyses (C,D) were expressed as means ± SE. Statistically significant in relation to *WT+SAL (*p*<0.05), #WT+BSA (*p*<0.05), ^+^5-LO^–/–^+SAL (*p*<0.05).

We then investigated the role of 5-LO products in the macrophage infiltration induced by BSA challenge [26]. Immunohistochemistry for the mouse macrophage antigen F4/80 revealed that BSA treatment induced cortical and medullary tubulointerstitial macrophage infiltration in the WT group (Figure 7A, C and B, D). Cortical and medullary macrophage infiltration was less pronounced in the 5-LO^–/–^+BSA group than in the WT+BSA group.

### Renal Cytokine Production

**Figure 8:**
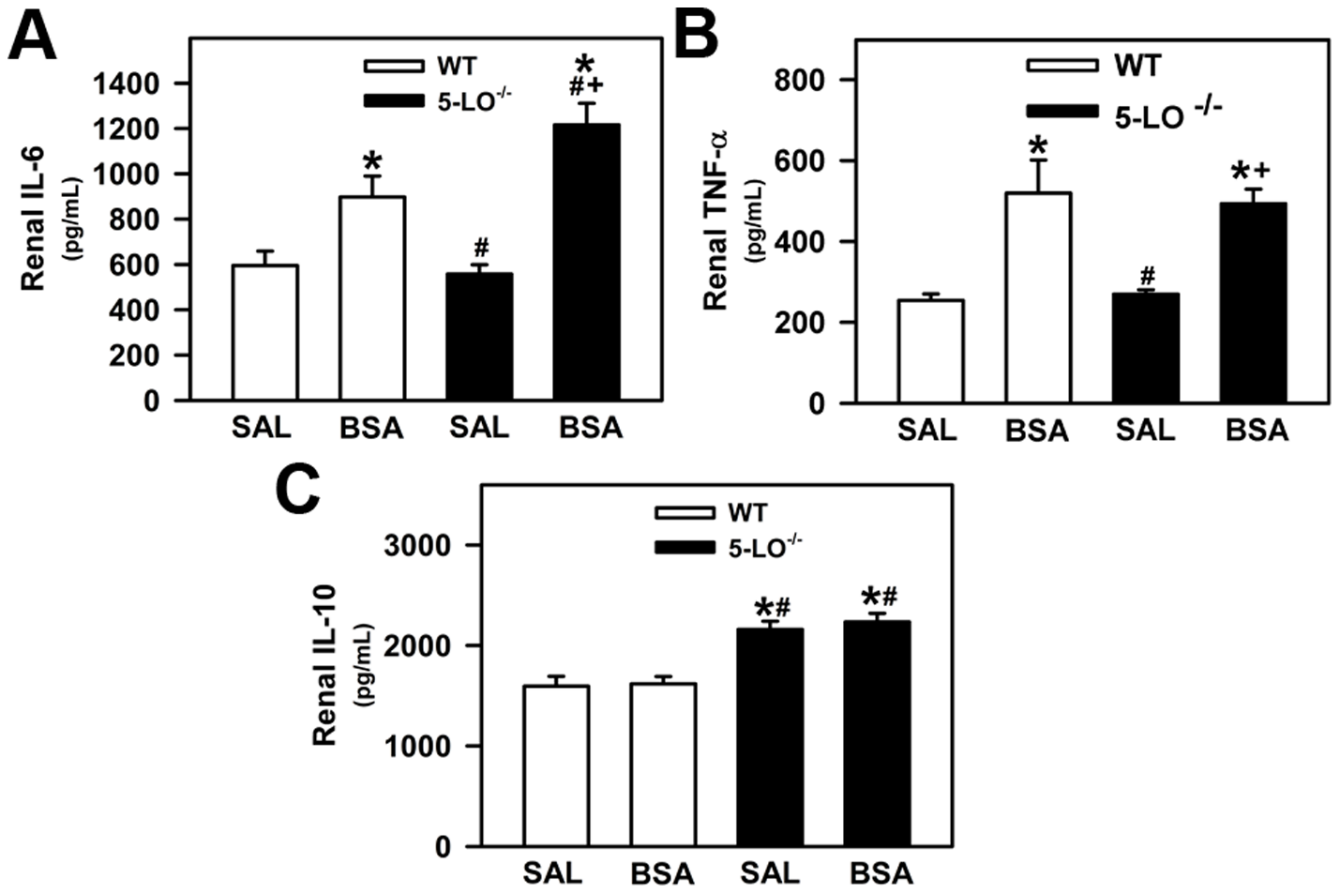
Analysis of renal cytokines in WT and 5-LO-deficient mice after saline or BSA treatments. (A) Renal IL-6, (B) TNF-α, and (C) IL-10 levels were determined by ELISA. The results are expressed as means ± SE. Statistically significant in relation to *WT+SAL (*p*<0.05), #WT+BSA (*p*<0.05), ^+^5-LO^–/–^+SAL (*p*<0.05).

**Figure 9:**
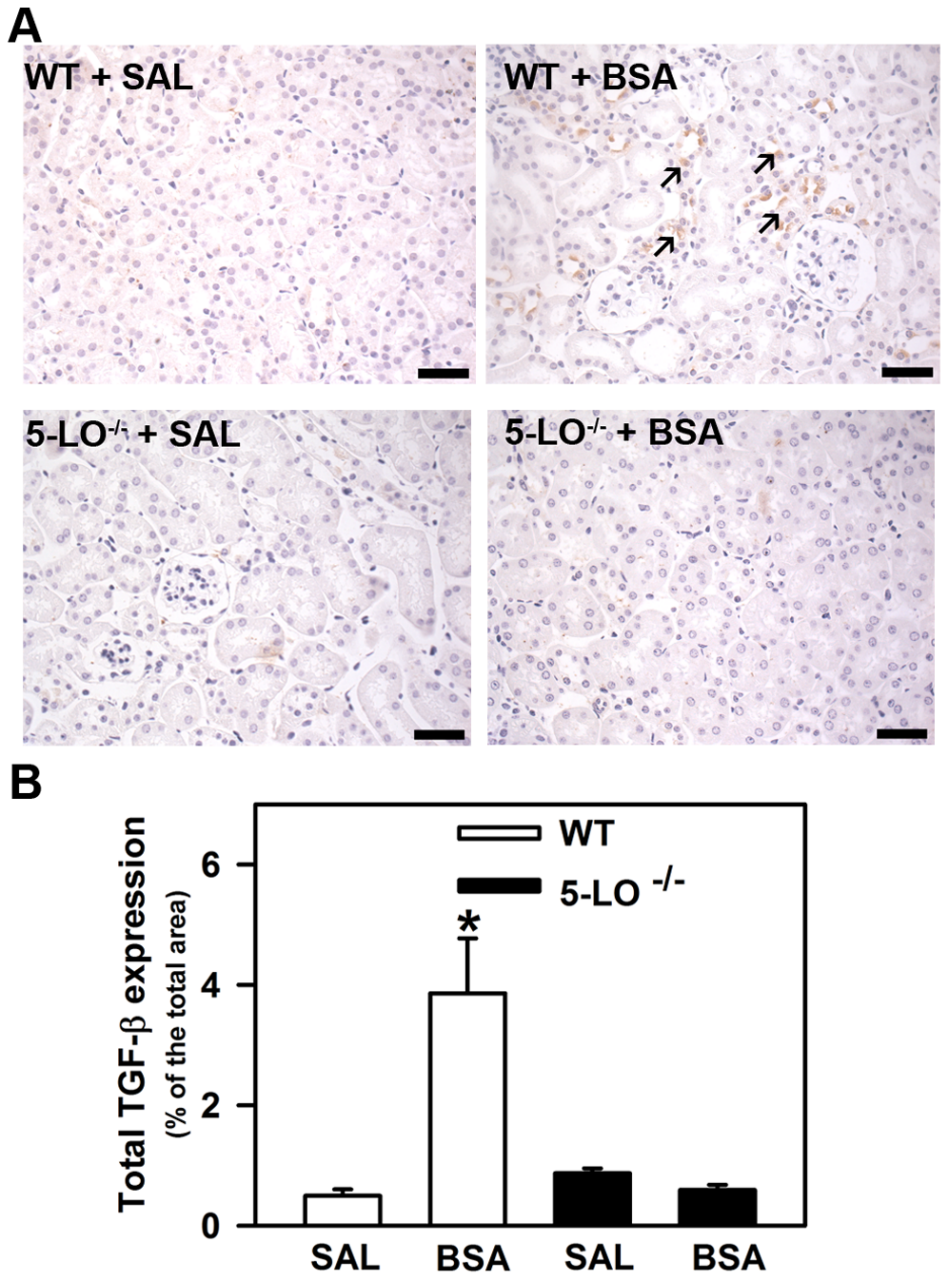
Effect of 5-LO products on the total cortical TGF-β expression increased during kidney injury. Mice were treated as described in Materials and Methods section (*n* = 6 per group). (A) Representative immunohistochemical staining for total TGF-β in cortical areas of WT mice and 5-LO-deficient mice treated with saline or BSA (bars  = 40 µm). Arrows indicates markedly positive staining. (B) Quantitative analyses were expressed as means ± SE. Statistically significant in relation to *WT + SAL (*p*<0.05).

Previous studies have shown that products of 5-LO are potent proinflammatory mediators by modulation of several cytokines and growth factors involved in the renal proinflammatory response [7]. In this context, we analyzed the level of IL-6, TNF-α, and IL-10. Renal levels of proinflammatory cytokines IL-6 and TNF-α were increased in WT+BSA and 5-LO^–/–^+BSA mice compared with WT+SAL and 5-LO^–/–^+SAL mice (Figure 8A, B, respectively). 5-LO^–/–^+BSA mice presented a further increase in the level of IL-6 compared with WT + BSA mice (Figure 8A). The level of anti-inflammatory cytokine IL-10 was significantly increased in the 5-LO^–/–^+SAL and 5-LO^–/–^+BSA groups compared with the respective WT groups (Figure 8C). These results show the possible involvement of IL-10 to ameliorate the interstitial injury observed in the 5-LO^–/–^+BSA group. In addition, total TGF-β expression was determined by immunohistochemistry for mouse TGF-β antigen in renal cortical slices, and increased expression of TGF-β was found in the WT+BSA group (Figure 9). In agreement with the analysis of renal collagen deposition, TGF-β did not increase in the 5-LO^–/–^ animals subjected to tubulointerstitial injury (5-LO^–/–^+BSA) (Figure 9).

## Discussion

It is well established that 5-LO products plays an important role in glomerular diseases, but their role in tubulointerstitial injury has still to be determined [13], [37]–[39]. The results obtained in the present work support the view that 5-LO products are also involved in tubulointerstitial injury induced by albumin overload in PTs. The effects of LTs in this process involve modulation of albumin endocytosis in PT cells, modulation of anti-inflammatory cytokine IL-10 production, TGF-β level, collagen deposition and macrophage infiltration. These results open new perspectives in understanding the role of LTs in the pathophysiologic mechanisms mediating progression of renal disease.

The progression of renal disease involves several factors including tubulointerstitial injury induced by albumin overload in PTs [20], [22]. Nowadays, it is accepted that a decrease in proteinuria is crucial in the treatment of different nephropathies [20]. However, the molecular mechanism involved in this process is still poorly understood make the choice of therapy difficult.

In the present work, we used an animal model of tubulointerstitial injury induced by BSA [25], [26]. This animal model is useful because there are no changes in the glomerular filtration rate (GFR) but albumin overload in PTs is observed. One important concern in the present study is the use of CCr as a marker of GFR. Hefler et al. [40] reported values for CCr in SV129 mice similar to those found in the present study. These values correlate very well with blood urea nitrogen levels. Similar results were also obtained by Andrea Babelova et al. [41] using a diabetic nephropathy model in the same mouse strain. In addition, they showed that CCr correlates very well with the level of serum cystatin C, a good marker of renal function. Goulet et al. [42], using clearance of inulin to measure the GFR, obtained values for the GFR in SV129 mice similar to those obtained with CCr. In a recent work, Schock-Kusch et al. [43] showed that the GFR, measured by fluorescent renal marker (FITC-Sinistrin) in SV129 mice is also similar to that obtained in the present study. Thus, it is plausible to postulate that the values of GFR measured by CCr in SV129 mice are not significantly different from those obtained by other techniques and therefore do not lead to misinterpretation of our results.

Proteinuria is a consequence of changes in protein filtration and/or endocytosis-mediated protein reabsorption in PT cells [20]. Usually, it is accepted that LTs are involved in proteinuria due to actions on glomeruli [38], [44]. Cysteinyl-LTs, such as LTD_4_, work as a mediator of proteinuria in passive Heymann nephritis [44]. This effect was associated with an increase in intraglomerular pressure. Valdivieso et al. [38], using a rat model of streptozotocin-induced diabetic nephropathy, a glomerulopathy model, also observed a correlation between expression of FLAP mRNA, LTs, and proteinuria. The authors suggested that LTs change the permeability of albumin in glomeruli without altering the estimated GFR. In agreement, Guasch et al. [39] observed that the oral administration of MK-591, a FLAP antagonist, restored the glomerular size selectivity without any changes in GFR in patients with glomerulonephritis, resulting in lower levels of proteinuria. We found that the proteinuria observed in the animal model of tubulointerstitial injury induced by BSA was lower in the 5-LO^–/–^ groups (5-LO^–/–^+SAL or 5-LO^–/–^+BSA) compared with the respective WT groups. The observations that GFR, cells per glomerulus, and glomerular nestin expression are not modified in the 5-LO^−/−^ groups indicates that LTs do not change protein filtration in the glomeruli. Furthermore, LTD_4_ and LTB_4_ inhibited the albumin uptake *in vitro* in PT cells. Thus, we suggest that the lower level of proteinuria in the 5-LO^–/–^ groups in this animal model is due to the increase in albumin endocytosis in PT cells rather than in albumin permeability as has been suggested for glomerulopathies.

How can 5-LO products modulate protein handling in PTs? It has been shown that activation of the PI-3K/PKB pathway increases albumin endocytosis, whereas the activation of PKC decreases it [33], [36], [45]. In agreement, we observed an increase in PKB activity and a decrease in PKC activity in 5-LO knockout mice. Furthermore, LTD_4_ and LTB_4_ decreased PKB activity and PKC inhibitor abolished the inhibitory effect of these LTs on albumin endocytosis. Therefore, our data indicate that 5-LO products decrease albumin endocytosis through inhibition of the PI-3K/PKB pathway and activation of PKC.

It is important to identify the link between modulation of albumin endocytosis by LTs and tubulointerstitial injury. It has been proposed that the sensor for variations in albumin concentration in the lumen of PTs is megalin [23]. Our group showed that a pathophysiologic albumin concentration decreases megalin expression leading to inhibition of the PI-3K/PKB pathway, which promotes the activation of endocytosis [23]. Similarly, it was observed in OK cells that albumin at pathologic concentration decreases its own endocytosis, which indicates a reduction in megalin expression [46]. Furthermore, under these conditions, albumin induces IL-8, IL-6, RANTES, and MCP-1 secretion in human PTE and LLC-PK1 cells [20], [23]. The observation that LTB4 and LTD4 decreased albumin endocytosis via inhibition of the PI-3K/PKB pathway, markers of megalin expression, indicate that LTs modulate megalin expression leading to secretion of proinflammatory cytokines.

Another important question is the possible role of NF-kB in tubule interstitial injury [47]–[51]. It has been shown that NF-κB activation leads to secretion of proinflammatory cytokines involved in tubule interstitial injury. 5-LO activates NF-κB through the generation of LTB_4_ intermediates, which involves an association between 5-LO and the NF-κB p65 subunit and its translocation to the nucleus [52]–[58]. In addition, it is known that activation of the PI-3K/PKB pathway leads to inhibition of NF-κB, a decrease in proinflammatory cytokines, and an increase in anti-inflammatory cytokines. In agreement, we observed that LTB4 and LTD4 inhibit PKB in LLC-PK1 cells and this is correlated to the increase in PKB activity observed in 5-LO^-/-^ animals. Some studies have shown that albumin at a pathophysiologic concentration increases NF-κB activity and inhibits PKB [20], [22], [59]. Thus, based on these observations, we propose that BSA-induced damage in tubular cells is likely also due to increased NF-κB activity through 5-LO activation and inhibition of the PI-3K/PKB pathway.

5-LO metabolites play an important role in acute and chronic inflammatory diseases [60]. Here, we observed that kidney insult in WT mice led to accumulation of renal IL-6, TNF-α, and macrophage infiltration. The observation that macrophage infiltration is decreased in 5-LO^−/−^+BSA groups compared with the WT groups indicate that the infiltrated immune cells decrease, which could led to lower production of proinflammatory cytokines. However, it was observed that the increase in TNF-α induced by kidney insult is not changed in 5-LO^−/−^ mice, indicating that this proinflammatory cytokine could be produced by other cells rather than by infiltrated immune cells. One possibility could be the secretion of cytokines by PT cells as already discussed. In agreement, we observed that the expression of TGF-β in cortical epithelial cells was lower in the 5-LO^–/–^+BSA group than in the WT+BSA group. This observation correlated with lower total collagen deposition in the 5-LO^–/–^+BSA group than in the WT+BSA group.

We observed that IL-6 levels in 5-LO^−/−^+BSA mice were increased in relation to WT+BSA mice. Endogenous IL-6 seems to have a dual role in tissue injury and the inflammation associated with renal injury. It has been accepted that although IL-6 is well recognized as a proinflammatory cytokine [61]–[64], it also acts as a regenerative or anti-inflammatory cytokine [64]–[71]. In the kidney, IL-6 shows an anti-inflammatory profile protecting the organ against ischemic/reperfusion (I/R) injury. It was demonstrated that enhanced formation of endogenous IL-6 mediates the protective effects of HMG-CoA reductase inhibitors in experimental renal I/R injury [68]. In sharp contrast with these observations, other studies have shown that IL-6 promotes renal injury, dysfunction, and inflammation during renal I/R injury and renal transplantation [61]–[63]. These dual effects indicate that IL-6 in renal injury promotes (1) an inflammatory response, through activation of its classic signaling pathway in immune cells, which exacerbates renal injury; (2) a protective response through its actions on epithelial tubular cells, which protects the kidney from further injury and maintains renal function [64]. Because epithelial tubular cells do not express IL-6 receptor (IL-6R), the protective effect of IL-6 in these cells could be mediated by a mechanism of trans-signaling, by which IL-6 stimulates target cells together with a soluble form of the IL-6R (sIL-6R) [64], [65]. During acute kidney injure (AKI), infiltrated neutrophils can release their membrane-bound IL-6R, increasing the levels of sIL-6R [72]. The molecular mechanism of such a protective effect involves gp130 and STAT3 activation by IL-6/sIL-6R in the renal epithelial cells, which leads to reduced lipid peroxidation and apoptosis, protecting the kidney against oxidative stress and further injury in the surrounding tissue [64], [65].

Another important point is the increased IL-10 level in 5-LO^−/−^ mice. It has been shown in different models of glomerulonephritis that increased IL-10 promotes deposition of mesangial immune complexes, glomerular mesangial cell proliferation, and albuminuria, which aggravates kidney injury [73]–[76]. Conversely, other studies showed that IL-10, even during glomerulonephritis and 5/6 nephrectomy, could have a protective effect. The possible mechanisms involved are inhibition of renal interstitial immune cell infiltration, decreased renal production of MCP-1, RANTES, and collagen type I and III [77]–[80]. These effects were associated with less proteinuria, decreased glomerulosclerosis and interstitial fibrosis [80]. Therefore, it is possible to postulate that the upregulation of IL-10 is crucial to determine the level of tubulointerstitial injury.

In the present work, we observed an increase in the level of IL-10 in the 5-LO^−/−^+SAL and 5-LO^−/−^+BSA groups, associated with increased IL-6 production after challenging with BSA, without significant alterations in the levels of TNF-α in relation to the WT controls, which indicates that the upregulation of IL-6 and IL-10 is directly due to the lack of 5-LO products. This phenomenon is associated with less proteinuria, reduced urinary LDH activity as well as decreased renal interstitial macrophage infiltration in 5-LO^−/−^+BSA mice. These results indicate that in our BSA-challenged tubular injury model, the high level of production of IL-6 and IL-10 in the 5-LO^−/−^ mice has a tubular protective effect. In agreement with this, we observed that the severity of tubulointerstitial injury induced by albumin overload in a sepsis animal model is also correlated to the level of IL-10 [26]. In addition, at the molecular level, it has been observed that PI3K/PKB pathway activation is associated with increased production of both IL-6 and IL-10 [81]. In agreement, we observed that BSA-challenged 5-LO^−/−^ mice showed increased PKB phosphorylation and higher levels of IL-6 and IL-10.

Our results indicate that LTs mediate, at least in part, tubulointerstitial injury induced by albumin overload, which could be important in the progression of renal disease.
